# OP78 A Consumer-Led, Co-Designed Process For Enhanced Consumer Engagement In The Australian Health Technology Assessment System

**DOI:** 10.1017/S0266462325101323

**Published:** 2025-12-29

**Authors:** Jo Watson, Liz Marshall, Rebecca Pitman, Sharon Winton, Alicia Segrave

## Abstract

**Introduction:**

The Australian Government Department of Health and Aged Care (Department) and Medicines Australia (the peak body for the pharmaceutical industry in Australia) have entered into a five-year strategic agreement. A core objective of this agreement was to develop a consumer-led, co-designed process to enhance consumer engagement earlier in the Australian health technology assessment (HTA) system.

**Methods:**

A co-design proposal was developed by a consumer reference group and included guidance on “consumer-led” processes such as establishing a Co-design Working Group (Group) of consumer, industry, and Department members weighted toward a greater number of consumers. Selection of consumer members was by a consumer-peer selection panel. An independent facilitator led the co-design process, founded on a work schedule and design principles developed by the Group. Recent consultation reports on consumer engagement in HTA processes guided the Group and enabled it to be “solution focused.” The Group sought open consultation on draft recommendations prior to finalizing a report of recommendations.

**Results:**

The Enhance HTA report describes recommendations for consideration by the Australian Minister for Health and Aged Care. The Group established a vision where “Australians’ diverse health care experiences and needs are understood, and consumer engagement is integral in HTA decision-making.” Enhance HTA includes 10 recommendations related to themes of partnerships, transparency, and evidence and input. The report notes the need for alignment with reformative HTA policies and methods, investment in systemic change, and development of a framework to embed the proposed recommendations along the health technology pathway. The co-design process and outcomes will be discussed if accepted for presentation.

**Conclusions:**

The consumer-led, co-designed process is a strong signal of the desire to engage consumers earlier in Australian HTA processes and in a more meaningful way. The expectations are high that the recommendations emerging from this process may be implemented to further embed consumer engagement into HTA processes, ensuring the diverse healthcare needs of Australians are integral to HTA decision-making.

